# Transcriptomic Data Sets To Determine Gene Expression Changes Mediated by the Presence of PBT2 in Growth Medium of Multidrug-Resistant Neisseria gonorrhoeae WHO Z

**DOI:** 10.1128/MRA.00283-20

**Published:** 2020-05-21

**Authors:** Freda E.-C. Jen, Ibrahim M. El-Deeb, John M. Atack, Mark von Itzstein, Michael P. Jennings

**Affiliations:** a Institute for Glycomics, Griffith University, Gold Coast, QLD, Australia; University of Maryland School of Medicine

## Abstract

Neisseria gonorrhoeae causes the sexually transmitted infection gonorrhea. High-coverage (∼3,300-fold) transcriptome sequencing data have been collected from multidrug-resistant N. gonorrhoeae strain WHO Z grown in the presence and absence of PBT2.

## ANNOUNCEMENT

Neisseria gonorrhoeae is a Gram-negative diplococcal bacterium that infects human mucosal surfaces and causes the sexually transmitted infection gonorrhea. Symptomatic gonococcal infections typically present as urethritis in males and cervicitis in females. Up to 80% of female gonococcal infections are asymptomatic (1, 2). Untreated or undetected infections can lead to pelvic inflammatory disease, infertility, and neonatal blindness; more importantly, infection is associated with increased HIV transmission (reviewed by Edwards et al. [3]). No vaccine is available, and the emergence of multidrug-resistant (MDR) N. gonorrhoeae strains that are resistant to available antimicrobials is a current health emergency (4). N. gonorrhoeae WHO Z, also called strain A8806, was identified in Australia in 2013 (5). WHO Z is resistant to penicillin G, cefixime, ceftriaxone, azithromycin, ciprofloxacin, and tetracycline, and it carries most known resistance genes (4).

PBT2 {5,7-dichloro-2-[(dimethylamino)methyl]quinolin-8-ol} is a hydroxyquinoline-based ionophore that was developed as a treatment for Alzheimer’s disease and Huntington’s disease, and it progressed to phase 2 human clinical trials (6). Recent studies showed that PBT2-zinc complexes can sensitize bacteria to antibiotics and can reverse antibiotic resistance in multiple Gram-positive bacteria (7) and in the Gram-negative pathogen N. gonorrhoeae (7, 8). To understand how PBT2 sensitizes N. gonorrhoeae to antibiotics, the transcriptome of strain WHO Z was determined in the presence and absence of 0.5 μM PBT2.

Cultures of strain WHO Z were grown to mid-log phase in GC broth before supplementation with PBT2 or dimethyl sulfoxide (DMSO) (the solvent for PBT2, as a no-PBT2 control) and then grown for an additional 16 h at 37°C. Triplicate biological replicates of total RNA (three separate cultures grown for each RNA sample) were extracted using TRIzol (Thermo Fisher Scientific) according to the manufacturer’s protocol. Libraries were prepared using the Illumina Ribo-Zero Gold protocol and were assessed using an Agilent Bioanalyzer DNA 1000 chip. Each individual library was quantified by quantitative real-time PCR and normalized to 2 nM by using the Illumina cBot system with TruSeq PE cluster kit v3 reagents. Sequencing of 150-bp paired-end runs was performed on the Illumina NovaSeq system with TruSeq SBS kit v3 reagents. The sequence reads from all of the samples were analyzed according to Australian Genome Research Facility (AGRF) quality control measures. The per-base sequence quality for the samples was excellent, with >93% of bases having a score above Q30 across all samples. The reads were also screened for the presence of any Illumina adapter or overrepresented sequences and cross-species contamination. The average number of reads for all samples was 98,389,274 reads (see details in Table 1). Sequence reads were aligned with the WHO Z reference genome (GenBank accession number GCF_900087715.2) by using Bowtie 2 aligner v2.3.3.1 with default settings (9). Transcripts were assembled using StringTie v1.3.3 (10). Counts were summarized at the gene level by featureCounts v1.5.3 (11). Default settings were applied in all software programs except where otherwise specified. Differences in gene expression due to the presence of PBT2 were expressed as log_2_(fold change). Analysis of log(counts/million) values was performed by averaging log(counts/million) across all samples. *F* values were calculated as the quasi-likelihood *F* statistic. *P* values were calculated to test for statistically different expressions, and false discovery rate-adjusted *P* values were calculated for multiple hypothesis testing. These new assembled transcriptomic data were used to determine differential gene expression influenced by the presence of PBT2 and to elucidate the mode of action of PBT2 in breaking antibiotic resistance in N. gonorrhoeae.

**TABLE 1:** Summary of sequencing reads and SRA accession numbers for RNA samples

Sample	Accession no.	No. of reads	% of reads mapped to genome	% of reads mapped to rRNA
DMSO_1	SRX7874779	98,161,838	88.50	<10
DMSO_2	SRX7874780	95,396,518	89.57	<10
DMSO_3	SRX7874781	82,450,694	89.41	<10
PBT2_1	SRX7874782	102,875,880	87.71	<10
PBT2_2	SRX7874783	117,431,706	88.56	<10
PBT2_3	SRX7874784	94,019,008	89.12	<10

### Data availability.

The GEO data set is available under accession number GSE146622. SRA accession numbers are provided in Table 1.
